# Role of Interleukin-10 on Nasal Polypogenesis in Patients with Chronic Rhinosinusitis with Nasal Polyps

**DOI:** 10.1371/journal.pone.0161013

**Published:** 2016-09-01

**Authors:** Jun Xu, Ruining Han, Dae Woo Kim, Ji-Hun Mo, Yongde Jin, Ki-Sang Rha, Yong Min Kim

**Affiliations:** 1 Department of Otorhinolaryngology-Head and Neck Surgery, Research Institute for Medical Science, Chungnam National University School of Medicine, Daejeon, Korea; 2 Department of Otorhinolaryngology-Head and Neck Surgery, Boramae Medical Center, Seoul National University College of Medicine, Seoul, Korea; 3 Department of Otorhinolaryngology-Head and Neck Surgery, Dankook University College of Medicine, Chonan, Korea; 4 Department of Otorhinolaryngology-Head and Neck Surgery, Yanbian University Hospital, Yanji, China; University of Pittsburgh, UNITED STATES

## Abstract

**Background and Objectives:**

Interleukin 10 (IL-10) is a potent anti-inflammatory cytokine. The dysregulation of IL-10 is associated with an enhanced immunopathologic response to infection, as well as with an increased risk for developing numerous autoimmune diseases. In this study, we investigated IL-10 expression in chronic rhinosinusitis with nasal polyps (CRSwNP) and assessed the possible role of IL-10 in the pathogenesis of CRSwNP.

**Materials and Methods:**

Thirty-five patients with CRSwNP, 12 patients with chronic rhinosinusitis without NP (CRSsNP) and 10 control subjects were enrolled in this study. NP tissues and uncinated tissues (UT) were collected for analysis. Dispersed NP cells (DNPCs) were cultured in the presence or absence of IL-25 and IL-10, and a flow cytometric assay was performed to identify the constitutive cell populations of the DNPCs. Murine NP (*n* = 18) models were used for the in vivo experiments. Real-time PCR, immunohistochemistry, western blotting analysis and ELISA were performed to measure the expression levels of the selected inflammatory cytokines and inflammation-associated molecules.

**Results:**

The mRNA expression levels of IL-10, IL-5, IL-17A, IL-25 and interferon gamma (IFN-γ) were significantly higher in the NP tissues than in the UT tissues. Strong positive correlations were observed between IL-10 and a variety of inflammatory cytokines (IL-5, IL-17A, IL-25, IFN-γ) and inflammation-associated molecules (B-cell activating factor; BAFF, CD19). Other than the IL-25 to IL-10 ratio, the expression ratios of the other measured inflammatory cytokines to IL-10 were significantly lower in the CRSwNP group than in the CRSsNP or control groups. Administrating IL-25 into the cultured DNPCs significantly increased the production of IL-10, but administrating IL-10 had no effect on the production of IL-25.

**Conclusion:**

Increased expression of IL-10, IL-10 related inflammatory cytokine, and IL-10 related B cell activation indicated that IL-10, a potent anti-inflammatory cytokine, has a pivotal role in the pathogenesis of CRSwNPs.

## Introduction

Chronic rhinosinusitis with nasal polyposis (CRSwNP) is a chronic inflammatory disease of the paranasal sinuses that leads to nasal obstruction, olfactory dysfunction, headaches, and posterior nasal drip, all of which can adversely affect a patient’s quality of life [1]. Cases of CRSwNP appear to be associated with a Th1-, Th2-, or Th17-biased inflammatory process, an increase in several pro-inflammatory and inflammatory cytokines, including interleukin 5 (IL-5), IL-13, IL-17A, interferon gamma (IFN-γ), IL-33 and IL-25 [2,3]. However, the underlying etiology of this severe inflammation may be multi-factorial, and the exact pathogenesis of CRSwNP is still unknown.

Effective control mechanisms are necessary for the immune response to eliminate the pathogens without causing damage to the host, and enhanced tissue transforming growth factor beta 1 (TGF-β1) and IL-10 expression by regulatory T cells have been known to play a crucial role in maintaining self-tolerance while preventing consumptive responses to the pathogens by suppressing the activity of pathogenic immune cells [4]. In light of this, there have been several studies that have investigated the role of regulatory T cells on the pathogenesis of CRSwNP by looking at their impaired function in suppressing severe inflammation [3,5–7]. However, their function in relation to polypogenesis is still not fully understood.

IL-10 is a potent anti-inflammatory cytokine that protects the host from excessive tissue damage during the host’s defense against pathogens and has a pivotal role in the development and maintenance of immune tolerance and homeostasis [8]. IL-10 is produced by numerous cell types, including T helper cells, monocytes, macrophages, dendritic cells, B cells, cytotoxic T cells, NK cells, mast cells, and granulocytes [8,9]. A deficiency of IL-10 is associated with the development of spontaneous colitis in mice and a number of autoimmune diseases [10,11]. In addition, impaired IL-10 expression or signaling can exaggerate the local inflammatory tissue response, resulting in exacerbated tissue immunopathology and tissue damage. Conversely, IL-10 expression is up-regulated in many chronic inflammatory diseases. A recent study demonstrated that pathogens simultaneously increase IL-10 secretion as well as the secretion of various inflammatory cytokines in dispersed NP cells and that these responses may play a role in the pathophysiology of CRSwNP [12]. To date, there is a large body of work that has evaluated IL-10 expression in patients with CRSwNP. Some of these have demonstrated increased expression of IL-10 in various types of CRS samples including serum [13], peripheral blood monocytes (PBMC) [14], nasal secretions [15] and nasal tissues [16–21], whereas others have reported opposite findings [22–30]. As a result, the role of IL-10 in the pathogenesis of CRSwNP is still subject to much debate. In this study, we have investigated IL-10 expression and its relation to other inflammatory cytokines in patients with CRSwNP in order to understand the role of IL-10 in the pathogenesis of CRSwNP.

## Materials and Methods

### Patients and tissue preparation

A total of 57 patients were enrolled in this study. Of the 57 patients, 35 had CRSwNP, 12 had CRS without NP (CRSsNP), while 10 subjects who were undergoing other rhinologic surgeries, such as skull base, dacriocystostomy, or endoscopic orbital decompression surgery were enrolled as control subjects. Uncinate tissues (UT) were obtained from the 35 patients with CRSwNP, 12 patients with CRSsNP, and 10 control subjects. Polyp tissues were obtained from the NP of patients with CRSwNP. NP tissues from 5 patients were used for the culture of dispersed NP cells (DNPCs), and those from 10 patients were used for flow cytometric analysis.

The NP tissues from the remaining subjects (20 patients) were cut into small pieces. Half of the samples were immediately soaked in TRIzol^@^ reagent (Invitrogen, Carlsbad, CA, USA) for downstream application, while the other half were fixed with 4% paraformaldehyde (Biosesang, Seongnam, Republic of Korea), embedded in paraffin, sectioned into 4 μm-thicknesses, mounted onto saline-coated micro slides (Muto Pure Chemicals, Tokyo, Japan), and subsequently used for our immunohistochemistry and immunofluorescence study. The remaining NP and UT tissues from the CRSwNP patients and UT tissues from the CRSsNP patients and control subjects were immediately soaked in MACS Tissue Storage Solution (Miltenyi Biotec, Bergisch Gladbach, Germany), which contained a protease inhibitor (Cell Signaling, Danvers, MA, USA) for downstream protein extraction.

A sinus disease diagnosis was based on patient history, clinical examination, nasal endoscopy, and computed tomography of the paranasal sinuses, as detailed by the guidelines contained in ‘‘EPOS 2012: European position paper on rhinosinusitis and NPs 2012” [31]. Patients who used oral or nasal corticosteroids or other medications (e.g., antibiotics or antileukotrienes) for 4 weeks before sample collection; those with recent upper respiratory tract infections; and patients undergoing revision were excluded from the study.

Details of the subjects’ characteristics are shown in Table 1. A written informed consent was obtained from each patient and control subject before enrollment into the study. The study was approved by the Institutional Review Board of the Chungnam National University Hospital.

**Table 1:** Patient characteristics.

Group	Controls	CRSsNP	CRSwNP
Total of subjects, *n*	10	12	35
Gender, male, *n* (%)	8 (80)	10 (83)	24 (68.6)
Age, median (IQR), year	39.5 (19.0)	44.0 (15.8)	53.0 (15.0)
Lund-Mackay CT score, median (IQR)	00 (00)	6.5 (4.3)	12.5 (4.3)

IQR = interquartile range.

### Culturing dispersed NP cells (DNPCs)

Dispersed NP cells (DNPCs) were prepared from NPs by means of enzymatic digestion using 2.0 mg/mL Protease from Streptomyces griseus (Sigma-Aldrich, St. Louis, MO, USA), 1.5 mg/mL Type 2 Collagenase (Worthington, Lakewood, NJ, USA), and 0.05 mg/mL Deoxyribonuclease I from bovine pancreas (Sigma-Aldrich, St. Louis, MO, USA), as previously described [32,33]. A gentleMACS^TM^ Dissociator (Miltenyi Biotec, Bergisch Gladbach, Germany) was used to dissociate the digested tissues into single-cell suspensions. The cell suspensions were then filtered through cell strainers (SPL, Pocheon, Republic of Korea) with a pore size of 70μm to remove any undigested tissue and then washed twice with a culture medium [RPMI-1640 Media (HyClone, Logan, Utah, USA) containing 10% fetal bovine serum, 100 IU/mL penicillin, 100 IU/mL streptomycin, and 1% L-glutamine]. The cell pellets were resuspended in ACK Lysing Buffer (Lonza, Walkersville, MD, USA) and washed twice with phosphate buffered saline (PBS). After washing, the DNPCs were suspended in the culture medium and plated in 12-well culture plates (Falcon, Franklin Lakes, NJ, USA) at a concentration of 2.5 × 10^6^ cells per well and cultured in the presence or absence of 100 ng/mL of rhIL-25 (R&D Systems, Minneapolis, MN, USA), and 100 ng/mL of rhIL-10 (R&D Systems, Minneapolis, MN, USA) at 37°C in a humidified atmosphere of 95% air and 5% CO_2_. The cells were harvested after 6 hours. The optimal time points and doses for maximum expression of IL-25 and IL-10 were determined before conducting the experiment.

### Murine NP model

#### Ethics statement

All animal experiments were given approval by the IACUC of Boramae Medical Center (No.2013-0001) and were performed under strict governmental and international guidelines on animal experimentation. Animal housing was done by professional animal keepers and all efforts were made to minimize suffering. Animals were housed at animal facilities of the Boramae Medical Center at Seoul National University. BALB/c mice were maintained in a specific pathogen free unit on a 12 hours light / 12 hours dark cycle with 30 minutes twilight period. The ambient temperature was 21±2°C, the humidity was 60±10% and the room air change is 20-fold. Mice were housed using a stocking density of 3–5 mice per cage. Mice were given water and ssniff R/M-H diet (ssniff Spezialdiäten GmbH, Soest, Germany) ad libitum.

The animal health status was checked daily for clinical signs, morbidity, and mortality. The mice would be euthanized if there was poor intake, respiratory distress or abdominal distension, or body weight loss more than 25% developed. Humane endpoint was not applied in this study due to that no animal death was observed in the experimental.

On the day of the experiments, mice were first anesthetized by inhalational isoflurane (Isoflurane® USP, Piramal Healthcare Limited, 502321 Andhra Pradesh, India) and then euthanized by CO2 narcosis and asphyxia followed by cervical dislocation at the end of the study.

#### Murine NP model and tissue preparation

A detailed experimental protocol for generating the mouse NP model was described in a previous article [2,34]. In brief, eighteen female BALB/c mice (4 weeks old, 20–25 g) (Damool Science, Daejeon, Korea) were divided into three groups: phosphate-buffered saline (PBS)-treated (control group, n = 6); allergic CRS model (3 months group, n = 6); and long-term allergic CRS model (6 months group, n = 6). In the control group, PBS was injected intraperitoneally and subsequently instilled intranasally. In the allergic CRS models, the mice were injected with 25 mg of OVA (Sigma, St Louis, Mo) in 2 mg of aluminum hydroxide gel administered intraperitoneally on days 0 and 5, followed by daily intranasal instillation of 3% OVA diluted in 40 μL of PBS from day 12 to day 17. Thereafter, the same amount of 3% OVA diluted in 40 μL of PBS was instilled 3 times a week from day 21 to day 102 (12 weeks, short-term group or 3 months group) or day 186 (24 weeks, long-term group or 6 months group). Intranasal instillation was performed in the head-down position, with the mouse’s head kept down for 30 seconds after instillation to prevent pulmonary provocation. In addition, the mice were challenged weekly with 10 ng of Staphylococcus aureus enterotoxin B (SEB; List Biologic Laboratories, Campbell, CA, USA) from day 49 through day 102 (8 weeks, short-term group) or day 186 (20 weeks, long-term group) after OVA instillation. The short-term mice were sacrificed on day 103, while the long-term and control mice were sacrificed on day 187 by CO_2_ asphyxiation. The mice were sacrificed 24 h after the last OVA or PBS challenge.

After exposing the nasal cavities of the mice, the nasal mucosae were meticulously excised under microscopic view using a small curette and micro-forceps. The harvested nasal mucosa was immediately soaked in TRIzol^@^ reagent for subsequent mRNA analysis. Nasal lavage was performed according to the method previously described [35]. Briefly, after partial tracheal resection under deep anesthesia, a micropipette was inserted into the posterior choana through the tracheal opening in the direction of the upper airway. Each nasal cavity was gently perfused with 200 μL PBS, and fluid from both nostrils was collected and centrifuged. The supernatants were stored at -80°C for downstream application.

### Real-time fluorescence quantitative polymerase chain reaction

Total RNA from the tissues and DNPCs was extracted using TRIzol^@^ reagent (Invitrogen, Carlsbad, CA, USA), according to the manufacturer’s instructions. A similar amount of tissues and cells was used for each subject. For cDNA synthesis, 1 μg of total RNA was transcribed with AccuPower^@^ RT PreMix (Bioneer, Daejeon, Republic of Korea), according to the manufacturer’s instructions. Polymerase chain reactions (PCR) were performed for cDNA synthesis using a T100^TM^ Thermal Cycler (Bio-Rad Laboratories, Hercules, CA, USA). The mRNA expression was analyzed using a CFX Connect^TM^ Real-Time PCR Detection System (Bio-Rad Laboratories, Hercules, CA, USA) with PowerUp^TM^ SYBR^@^ Green Master Mix (Applied Biosystems, Carlsbad, CA, USA). The sequences of the self-designed primers purchased from GenoTech (GenoTech, Daejeon, Republic of Korea) are presented in S1 Table.

All PCR assays were performed in triplicate. For each sample, the differences in threshold cycles between the target molecules and glyceraldehyde 3-phosphate dehydrogenase (GAPDH) (∆Ct_target gene_, ∆Ct_reference gene_) were determined; a calibrated delta Ct value (∆∆Ct, ∆Ct_target gene_ - ∆Ct_reference gene_) was calculated; and the relative quantitation (RQ) values were then calculated using the following equation: RQ = 2^-∆∆Ct^.

### Immunohistochemistry and immunofluorescence studies

The paraffin-embedded tissue samples were soaked first in xylene to remove the paraffin wax and then sequentially in solutions of 100%, 95%, and 70% ethanol for rehydration. Antigen retrieval was performed by heating the slides in a Decloaking Chamber (Biocare Medical, Concord, CA) to 120°C. A protein block [10% normal chicken serum (Vector, Burlingame, CA, USA) in PBS and 0.3% Triton X-100 (Biosesang, Seongnam, Republic of Korea) for 1 hour at room temperature] was then applied to the tissue to prevent non-specific protein binding.

For single immunostaining, endogenous peroxidase activity was blocked by incubating the sections in 1% hydrogen peroxide solution (Sigma-Aldrich, St. Louis, MO, USA) in PBS with 0.3% Triton X-100 for 30 minutes at room temperature. Goat anti-human IL-10 antibody (R&D Systems, Minneapolis, MN, USA), at a concentration of 10μg/mL, was used as the primary antibody which was incubated with the tissue at 4°C overnight. Negative controls were prepared using PBS in place of the primary antibody. The sections were rinsed three times with PBS and then incubated for 2 hours at room temperature with biotinylated rabbit anti-goat IgG (Vector, Burlingame, CA, USA) as a secondary antibody. After further washing, the slides were incubated in horseradish peroxidase streptavidin (Vector, Burlingame, CA, USA) before being stained with 3,3′-Diaminobenzidine tetrahydrochloride hydrate (Sigma-Aldrich, St. Louis, MO, USA). After rinsing with PBS, the samples were mounted using Permount^TM^ Mounting Medium (Fisher Chemical, Fair Lawn, NJ, USA). The slides were then observed under an optical microscope (Olympus, Tokyo, Japan). IL-10-positive cells were counted per high-powered field (HPF, × 400) at three different sites in the tissue and the mean values with range were calculated.

For single or double-immunofluorescence staining, the corresponding rabbit anti-staphylococcal protein A (SpA) antibody (Abcam, Cambridge, MA, USA), goat anti human IL-10 antibody (R&D Systems, Minneapolis, MN, USA), and rabbit anti human IL-25 antibody (Abcam, Cambridge, MA, USA) were applied and left overnight at 4°C. The negative controls were incubated with PBS in place of the primary antibody. The slides were rinsed 3 times with PBS before incubating for 2 hours with the appropriate secondary antibodies. Double immunofluorescence experiments were performed using biotinylated rabbit anti-goat IgG (Vector, Burlingame, CA, USA) and Cy™3-Streptavidin (GE Healthcare, Tokyo, Japan) at a dilution of 1:1000 for goat anti-human IL-10 antibody, and Alexa Fluor@ 488-conjugated goat anti-rabbit IgG (Life Technologies, Carlsbad, CA, USA) at a concentration of 10 μg/mL for rabbit anti-SpA antibody and rabbit anti human IL-25 antibody. After rinsing with PBS, 4',6-diamidino-2-phenylindole (DAPI; Invitrogen, Carlsbad, CA, USA) was used at a concentration of 300 nM for nuclear counterstaining. After final rinsing with PBS, the samples were mounted using Fluoro-Gel with Tris Buffer (Electron Microscopy Science, Hatfield, PA, USA). The slides were subsequently observed on a fluorescence microscope (Olympus, Tokyo, Japan).

### Western blotting analysis

After placing the tissues from the patients in RIPA buffer (Cell Signaling, Danvers, MA, USA) containing protease inhibitor, the tissues were minced using sterilized scissors. Then, the tissues were further ruptured by using the mixer mill MM 400 (Retsch, Haan, Germany) and centrifuged at 12,000 rpm for 15min. The supernatant was transferred to new tubes and quantified with the Bradford assay (Bio-Rad, Hercules, California, USA). 50 μg of proteins from the supernatant were denatured and separated by 10% sodium dodecyl sulfate-polyacrylamide gel electrophoresis and transferred to polyvinyl difluoride (PVDF) membranes (Millipore, Billerica, MA, USA). The blots were incubated in a blocking buffer containing 5% BSA in TBST buffer (20 mM Tris-base; 137 mM NaCl, pH 7.6; and 0.05% Tween 20) for 1 hour and incubated overnight with Human IL-10 Antibody (R&D Systems, Minneapolis, MN, USA) and β-Actin Antibody (Cell Signaling, Danvers, MA, USA) as primary antibodies. After washing 3 times in TBST buffer, the blots were incubated with horseradish peroxidase (HRP)-conjugated secondary antibodies (Cell Signaling, Danvers, MA, USA). The membranes were developed using a chemiluminescent reagent (ECL; Millipore, Billerica, MA, USA) and subsequently exposed to chemiluminescent film to visualize the proteins. β-actin was used as a control for protein loading.

### Enzyme-linked immunosorbent assay

Levels of IL-10 in the murine nasal lavage fluids were measured using enzyme-linked immunosorbent assay (ELISA) kits (BioLegend, San Diego, CA, USA) according to the manufacturer's instructions. The lower detection limit of these ELISA kits was 2.7 pg/mL.

### Flow cytometric assay

Cellular suspensions were obtained by following the protocol used to prepare DNPCs. Immunostaining was performed using monoclonal fluorochrome-conjugated antibodies (Miltenyi Biotec, Bergisch Gladbach, Gemany) for a variety of cell surface markers to identify the cellular components of DNPCs. The antibodies used for cell surface staining are presented in S2 Table. 7-Aminoactinomycin D (7-AAD) (Sigma-Aldrich, St. ILouis, MO, USA) was used to identify viable cells. Cell surface staining was done by incubating the cells with fluorochrome-conjugated antibodies for 10 minutes at 4°C in the dark. The cells were then washed twice with PBS and stained with 7-AAD (Sigma-Aldrich, St. Louis, MO, USA) for 20 minutes at 4°C in the dark. The cells were washed twice with PBS and fixed in 1% paraformaldehyde (Biosesang, Seongnam, Republic of Korea) for 30 minutes at 4°C in the dark. Again, the cells were washed twice with PBS and re-suspended. Multi-parameter flow cytometry was performed using a BD LSR II instrument (BD Biosciences, San Jose, CA, USA) using FACS Diva software (BD Biosciences, San Jose, CA, USA), and the flow cytometry data were analyzed with FlowJo software (Tree Star, Ashland, OR, USA). The frequency of DNPCs expressing cytokeratin (21.39 ± 2.69), vimentin (16.49 ± 5.87), CD15/Singlec-8 (7.1 ± 5.6), CD4 (12.6 ± 0.4), CD8 (7.18 ± 3.5), CD79a (6.98 ± 3.4), CD68 (9.46 ± 6.86), CD117 (2.9 ± 2.6), CD11c/CD303/human leukocyte antigen-antigen D related (HLA-DR) (5.46 ± 2.45), and CD1c/HLA-DR (CD11c^-^) (0.95 ± 0.08) indicated the presence of epithelial cells, fibroblasts/vascular endothelial cells, eosinophils, CD4^+^ T cells, CD8^+^ T cells, B cells, macrophages, mast cells, plasmacytoid dendritic cells, and myeloid dendritic cells, respectively (S1 Fig).

### Statistical analyses

Statistical analyses were performed using SPSS 22 (version 22.0.0.0, International Business Machines, Armonk, NY, USA) and GraphPad Prism 6 (version 6.01, GraphPad Software, La Jolla, CA, USA). For continuous variables, the results were shown as mean with standard error of mean (SEM). Differences among groups were analyzed by using *Kruskal-Wallis* tests (2-tailed). *Spearman*’s rank correlation coefficient was used to determine variable relationships. A *p* value of less than 0.05 was considered statistically significant.

## Results

### Expression of IL-10 was significantly up-regulated in patients with CRSwNP

**Fig 1:**
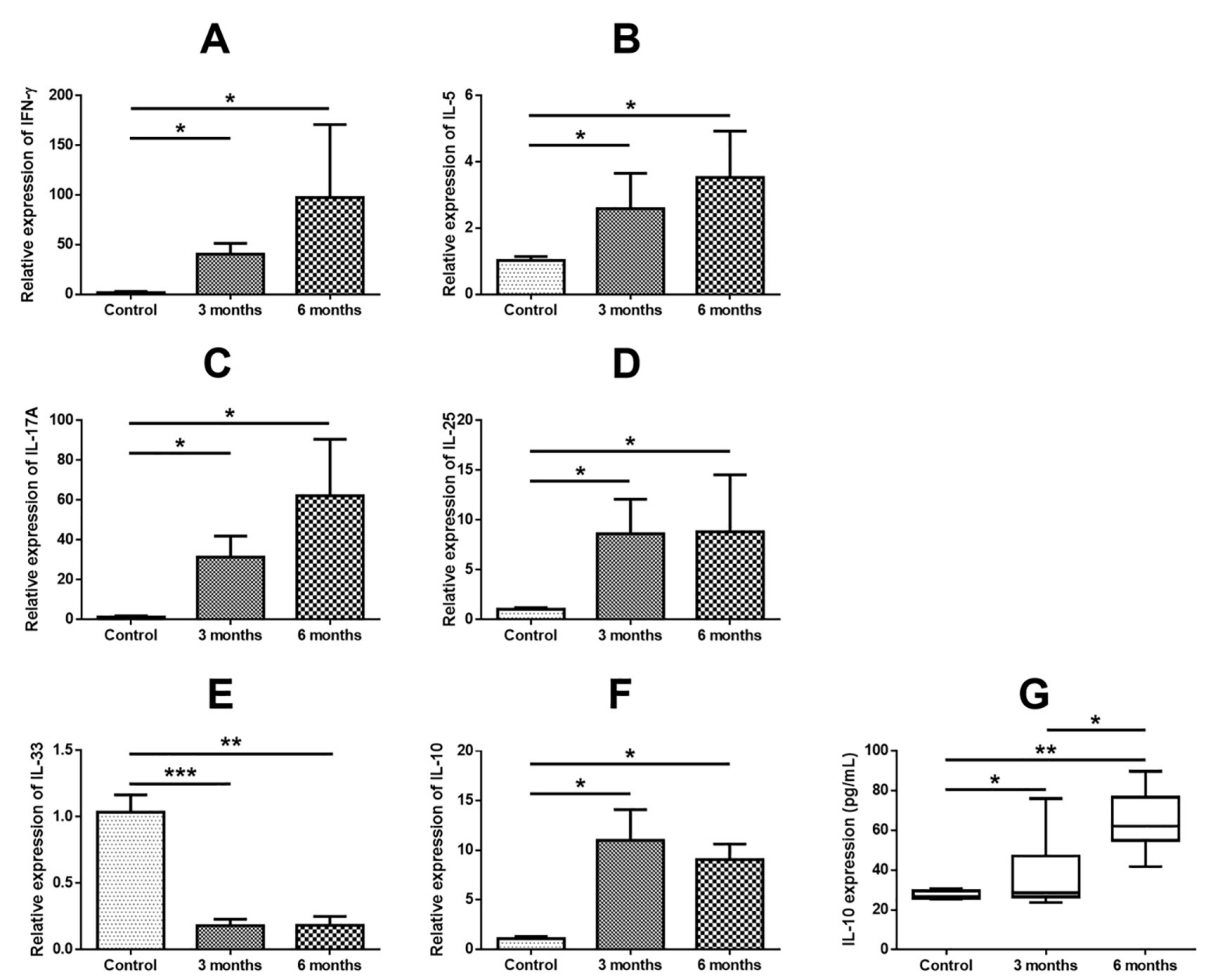
Expression of IL-10 among the different groups. A, mRNA expression of IL-10 in the UT tissue or NP tissue among groups. B, representative images of western blot assay and relative expression of IL-10 in the UT or NP among the groups. C, representative images of IL-10 immunohistochemistry and the number of IL-10 positive cells in the tissues from the different groups. CRSsNP, chronic rhinosinusitis without NP; CRSwNP, chronic rhinosinusitis with NP. * = *p*<0.05, ** = *p*<0.01, *** = *p*<0.001.

The mRNA expression of IL-10 was significantly higher in both the UT and NP of the CRSwNP group than in the UT of the control and the CRSsNP groups. It was also significantly higher in the UT of the CRSsNP group than in the control group (Fig 1A). The protein level of IL-10 which was assessed with the western blot analysis was also significantly higher in the NP tissues of the CRSwNP group than in the UT tissues of the control, CRSsNP, and CRSwNP groups (Fig 1B). Immunohistochemistry revealed that the number of IL-10 positive cells were significantly higher in the NP of the CRSwNP group than in the UT of the control, CRSsNP, and CRSwNP groups (Fig 1C). The number of IL-10 positive cells was also significantly higher in the UT of the CRSsNP and CRSwNP groups than in the UT of the control group (Fig 1C).

### Expression of T helper (Th) and innate cytokines among groups

**Fig 2:**
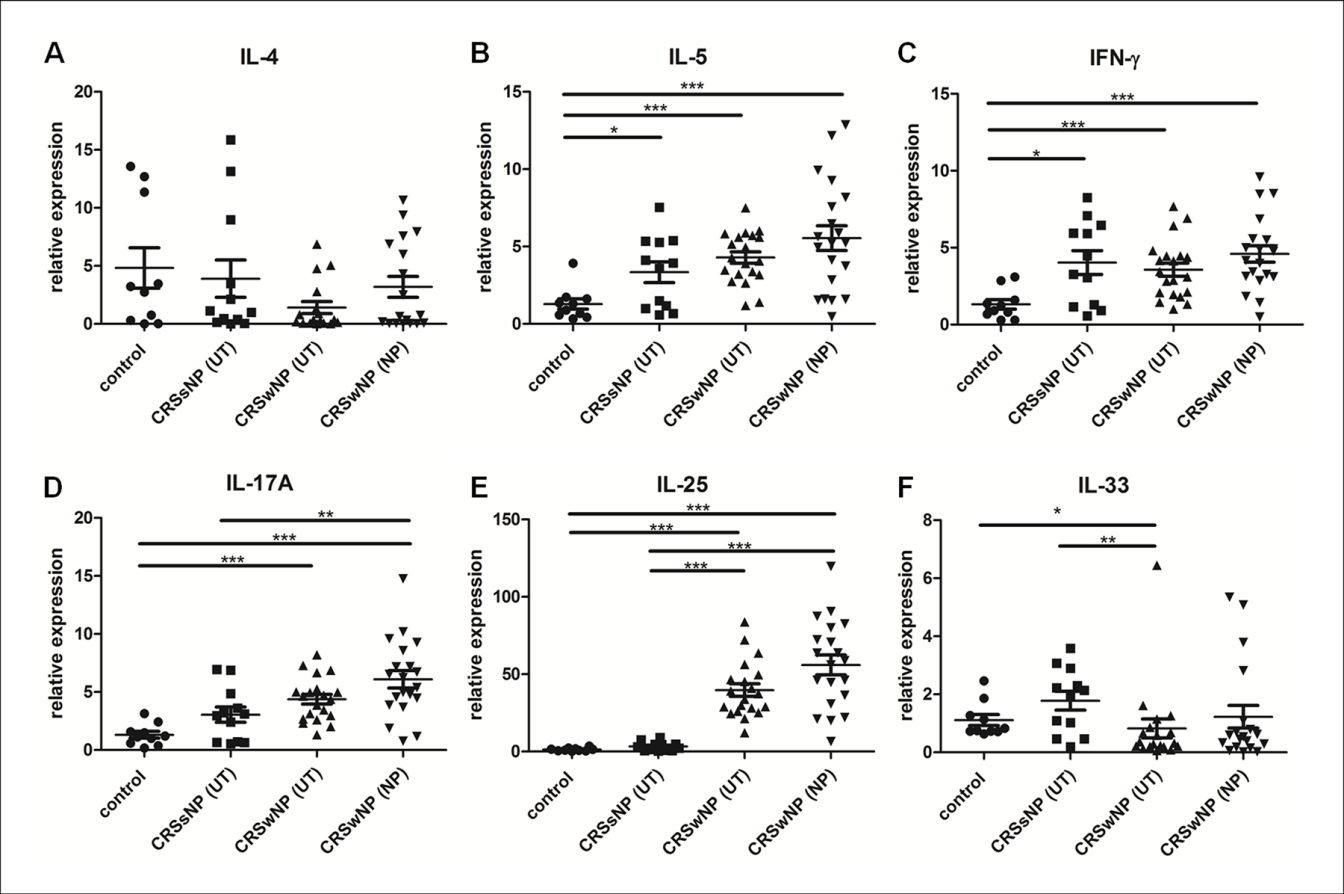
Expression of T helper and innate cytokines among the groups. mRNA expression of T helper (IL-4, IL-5, IL-17A, and IFN-γ) and innate (IL-25, and IL-33) cytokines using real time PCR (A to F) among the groups. * = *p*<0.05, ** = *p*<0.01, *** = *p*<0.001.

The mRNA expressions of selected cytokines (IL-4, IL-5, IL-17A, IFN-γ, IL-25, and IL-33) were assessed using real time PCR (Fig 2). Expressions of IL-5 and IFN-γ were significantly lower in the UT of the control group than in the UT of the CRSsNP group and in the UT and NP of the CRSwNP group (Fig 2B and 2C). Expression of IL-17A was significantly higher in the NP of the CRSwNP group than in the UT of the control and CRSsNP groups (Fig 2D). In addition, IL-17A was also significantly higher in the NP of the CRSwNP group than in the UT of the CRSsNP group (Fig 2D). The expression pattern of IL-25 was similar to that of IL-10 (Fig 1A) wherein the expression of IL-25 was significantly higher in both the UT and NP of the CRSwNP group than in the UT of the control and the CRSsNP groups (Fig 2E). Expression of IL-33 was significantly higher in the UT of the control and CRSsNP groups than in the UT of the CRSwNP group (Fig 2F).

Relative expression ratios of selected cytokines (IL-5, IFN-γ, IL-17A and IL-25) to IL-10 were calculated (S2 Fig). The expression ratio of IL-25 to IL-10 was significantly higher in both the UT and NP of the CRSwNP group than in the UT of the CRSsNP and the control groups (S2 Fig). However, the expression ratios of IL-5, IL-17A, and IFN-γ to IL-10 in the UT and NP of the CRSwNP group were all significantly lower than their corresponding ratios in the UT of the CRSsNP and the control groups (S2 Fig).

### Correlations of expression between IL-10 and inflammatory cytokines

**Fig 3:**
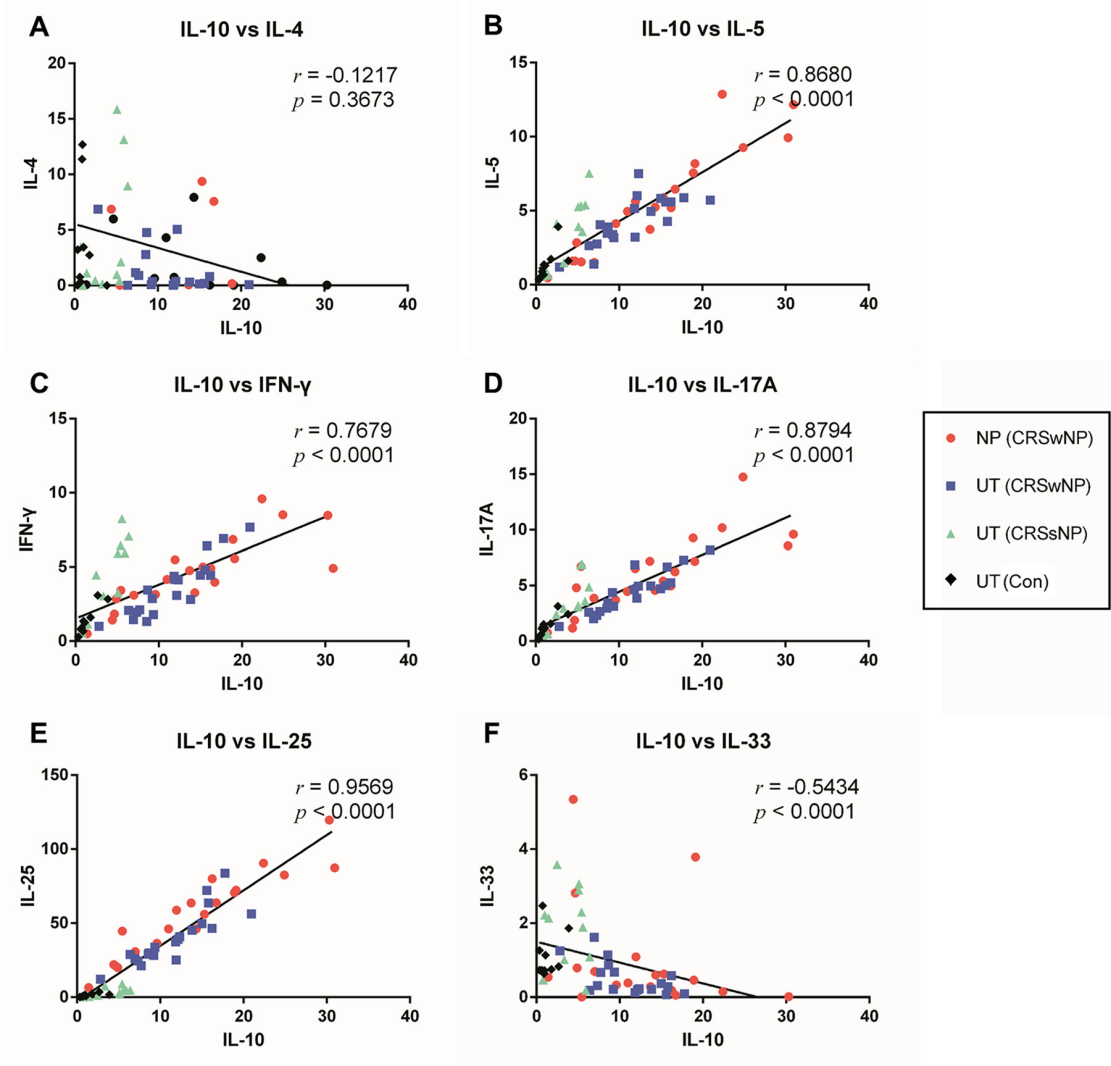
Correlations between IL-10 and inflammatory cytokine expressions in tissues from patients. Expression of IL-10 showed significant positive correlation with IL-5 (B), IFN-γ (C), IL-17A (D) and IL-25 (E) expressions in all patients. Expression of IL-10 and IL-33 showed significant negative correlation in all patients (F). There was no significant correlation between IL-10 and IL-4 (A). *r* = *Spearman*’s rank correlation coefficient.

Expressions of IL-10 showed significant positive correlation with those of IL-5, IFN-γ, IL-17A and IL-25 in all of the patients (Fig 3B–3E). There was an especially strong positive correlation between IL-10 and IL-25 (Fig 3E). The expressions of IL-10 and IL-33 showed significant negative correlation in all of the patients (Fig 3F). However, there was no significant correlation between the expressions of IL-10 and IL-4 (Fig 3A).

**Fig 4:**
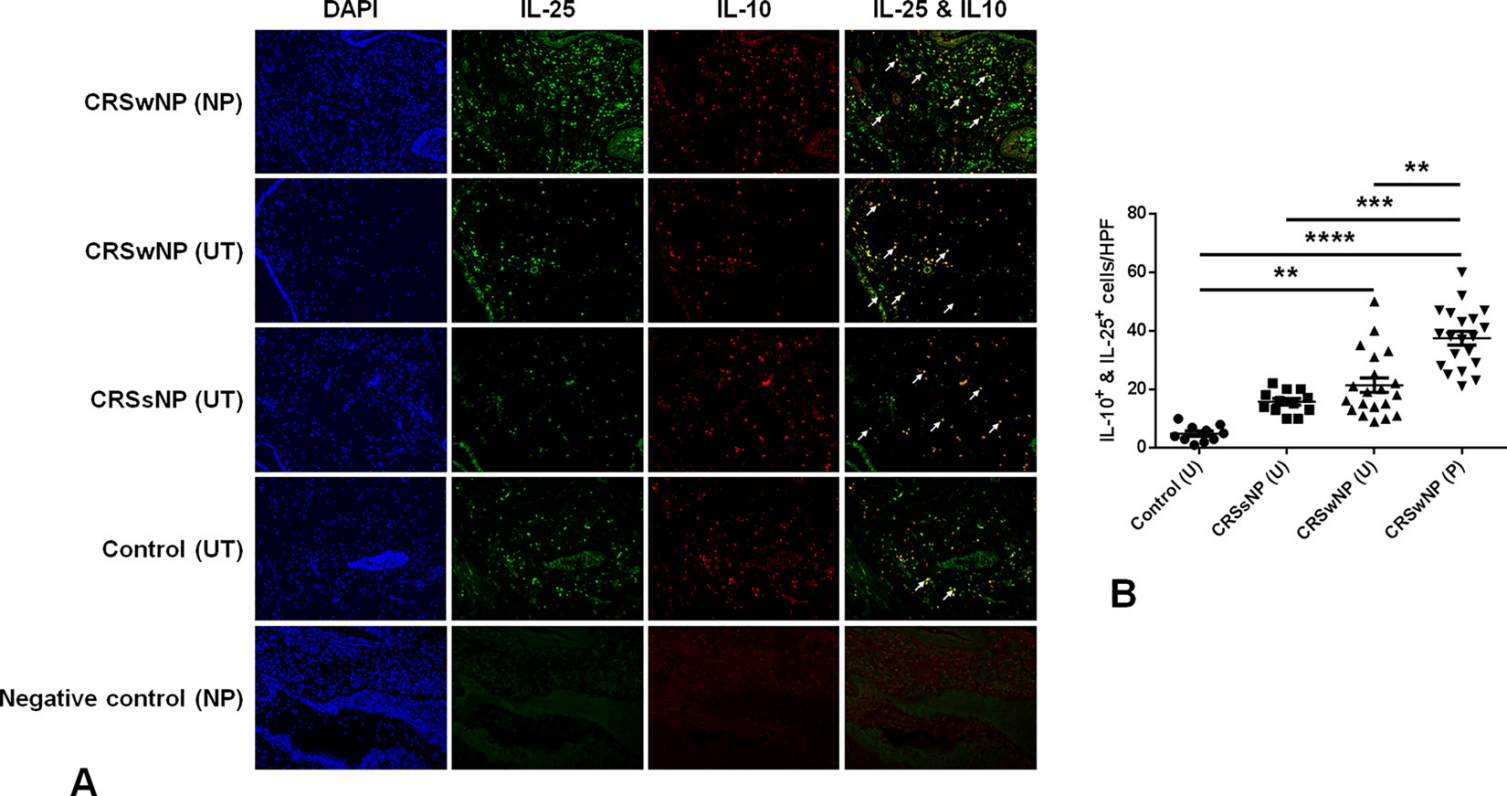
Double immunofluorescent staining of IL-10 and IL-25 in NP. Some of the cells in the subepithelial space of NP from patients with CRSwNP produced both IL-10 and IL-25 at the same time (A, white arrow). The number of double positive cells (IL-25^+^ IL-10^+^ cells) was considerably higher in the NP tissues of the CRSwNP group compared to the other groups and also much higher in the UT of the CRSwNP compared to the UT of the control group (B). ** = *p*<0.01, *** = *p*<0.001, **** = *p*<0.0001.

Because of the strong correlation between IL-10 and IL-25 and an increased IL-25 to IL-10 expression ratio, double immunofluorescent staining was conducted to investigate whether IL-10 and IL-25 were produced by the same cells in the tissue. Indeed, some cells in the subepithelial space of the NP and UT produced both IL-10 and IL-25 (Fig 4). The number of double positive cells (IL-25^+^ IL-10^+^ cells) was significantly higher in the NP tissues of the CRSwNP group compared to the other groups and also significantly higher in the UT of the CRSwNP group compared to the UT of the control group (Fig 4B).

### Correlations between Lund-Mackay CT scores and inflammatory cytokines

To evaluate the correlation between IL-10 and the severity of CRSwNP, Lund-Mackay CT scores were assessed in patients with CRSwNP. An evaluation of the CT scores and mRNA expression of IL-10 and other cytokines, including IL-25, IL-33, IFN-γ, IL-17A, IL-4, and IL-5 (S3 Fig) revealed significant positive correlations between the CT scores and expressions of other cytokines (IL-25, IFN-γ, IL-17A, and IL-5), as well as IL-10, in patients with CRSwNP.”

### Cytokine production in dispersed NP cells (DNPCs) culture

**Fig 5:**
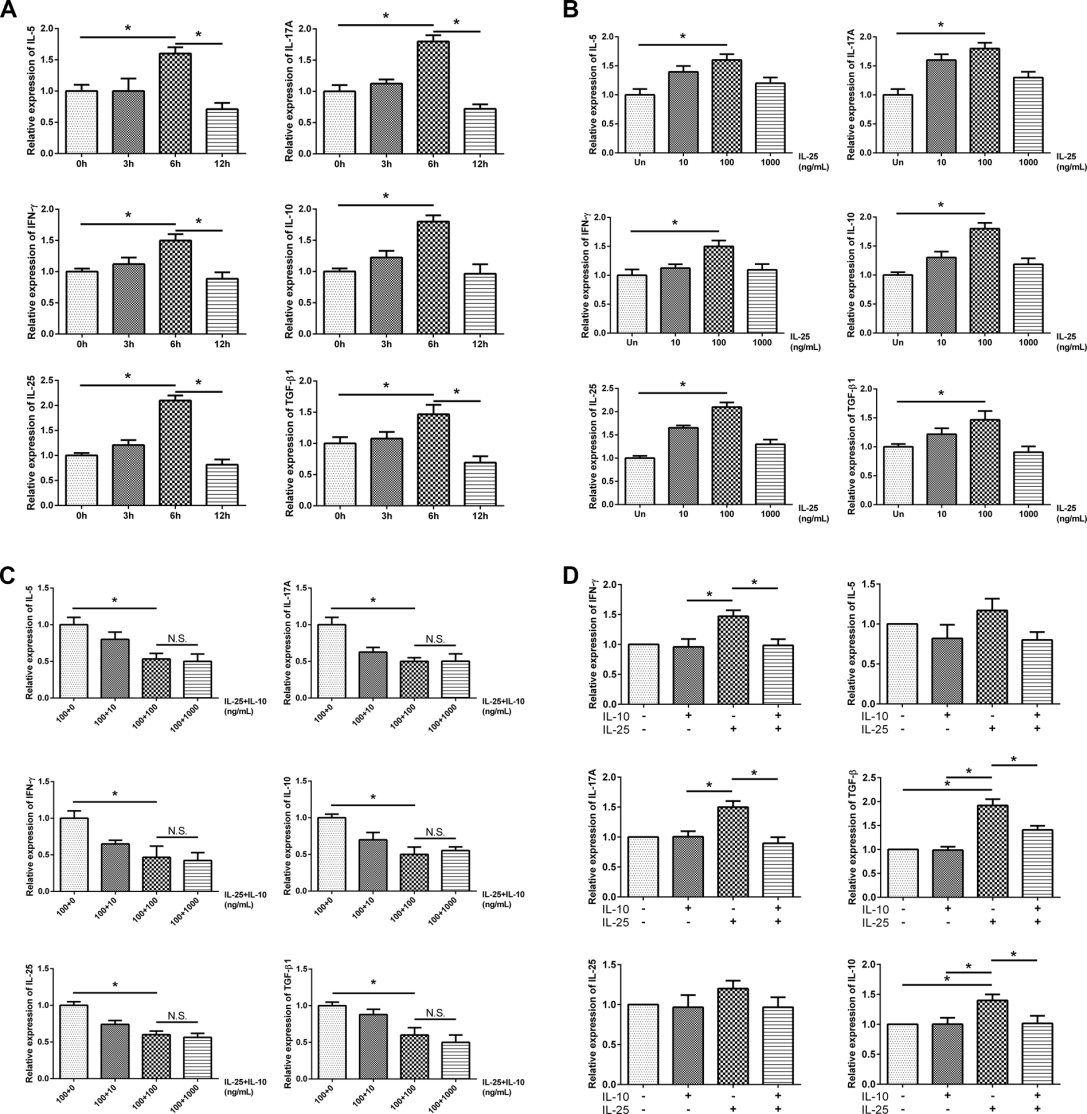
Cytokine production in dispersed NP cells (DNPCs) culture. The mRNA expression levels of IFN-γ, IL-5, IL-17A, IL-10, IL-25, and TGF-β1 became increased and peaked at 6 hours (A) after treatment with 100 ng/mL of IL-25 (B). Then, the cultured DNPCs were co-treated with 100 ng/mL of IL-25 and 10, 100 or 1,000 ng/mL of IL-10 for 6 hours (C). The mRNA expression levels of IFN-γ, IL-5, IL-17A, IL-10, IL-25, and TGF-β1 were most suppressed after treatment with 100 ng/mL of IL-10 (D). * = p<0.05, N.S. = not significant.

To determine the optimal treatment time point and dose of IL-25, the cultured DNPCs were treated with 10, 100 or 1,000 ng/mL of IL-25 for 0, 3, 6 or 12 hours (Fig 5A and 5B). The mRNA expression levels of IFN-γ, IL-5, IL-17A, IL-10, IL-25, and TGF-β1 became increased and peaked at 6 hours after treatment with 100 ng/mL IL-25 (Fig 5A). Then, the cultured DNPCs were co-treated with 100 ng/mL of IL-25 and 10, 100 or 1,000 ng/mL of IL-10 for 6 hours (Fig 5C). The mRNA expression levels of IFN-γ, IL-5, IL-17A, IL-10, IL-25, and TGF-β1 were most suppressed after treatment with 100 ng/mL of IL-10 (Fig 5D).

### Detection of Staphylococcal SpA

**Fig 6:**
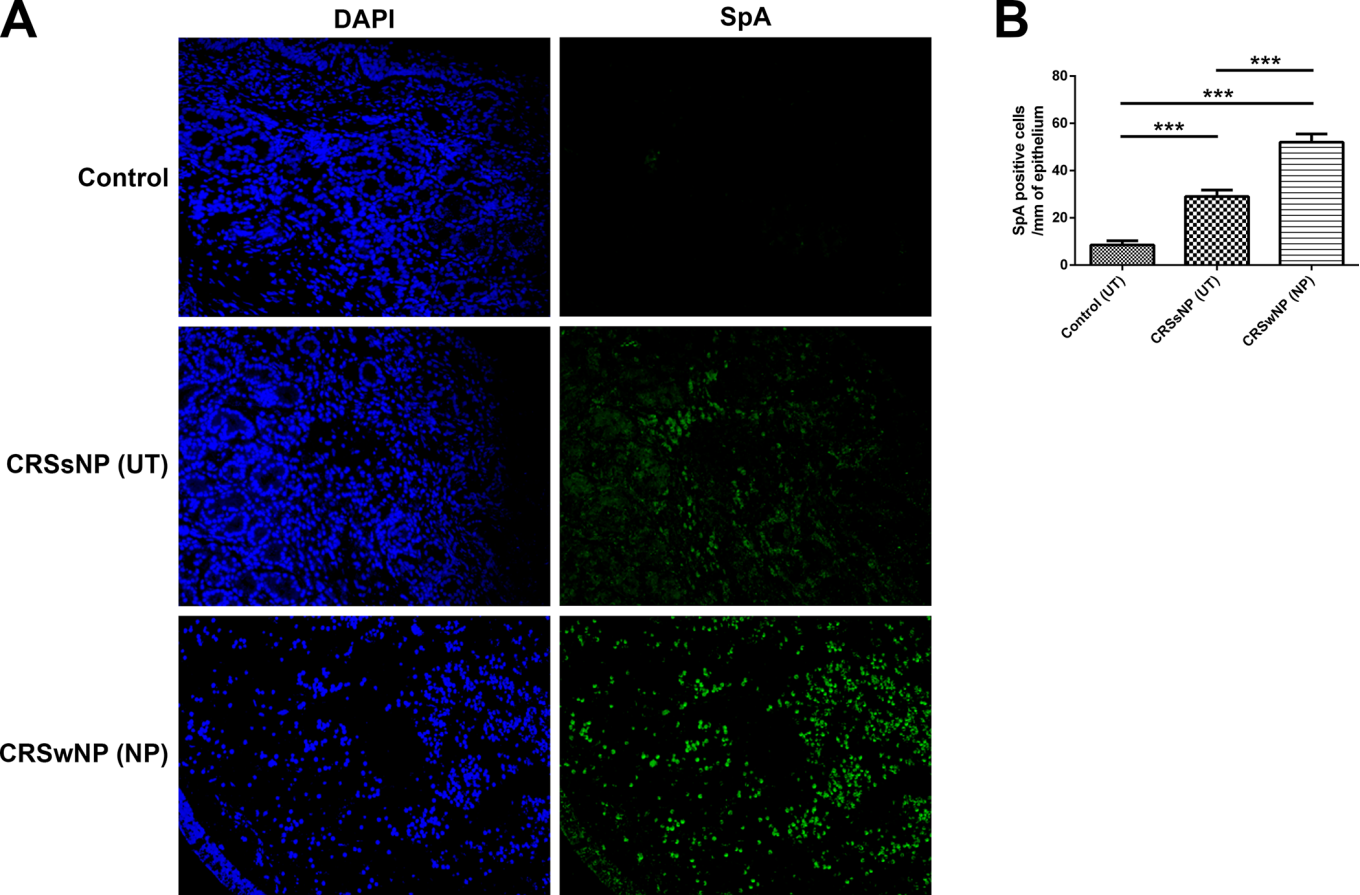
Detection of Staphylococcal SpA. A large amount of Staphylococcus SpA was detected in the NP of CRSwNP compared to the UT of the control group or patients with CRSsNP (Immunofluorescent staining A and B). * = *p*<0.05, ** = *p*<0.01, *** = *p*<0.001.

Immunofluorescent staining was conducted to detect Staphylococcus SpA in the tissue. A large amount of SpA was detected in the NP compared to the UT of the control or CRSsNP groups (Fig 6A and 6B).

### Influence of IL-10 expression on B cell proliferation

**Fig 7:**
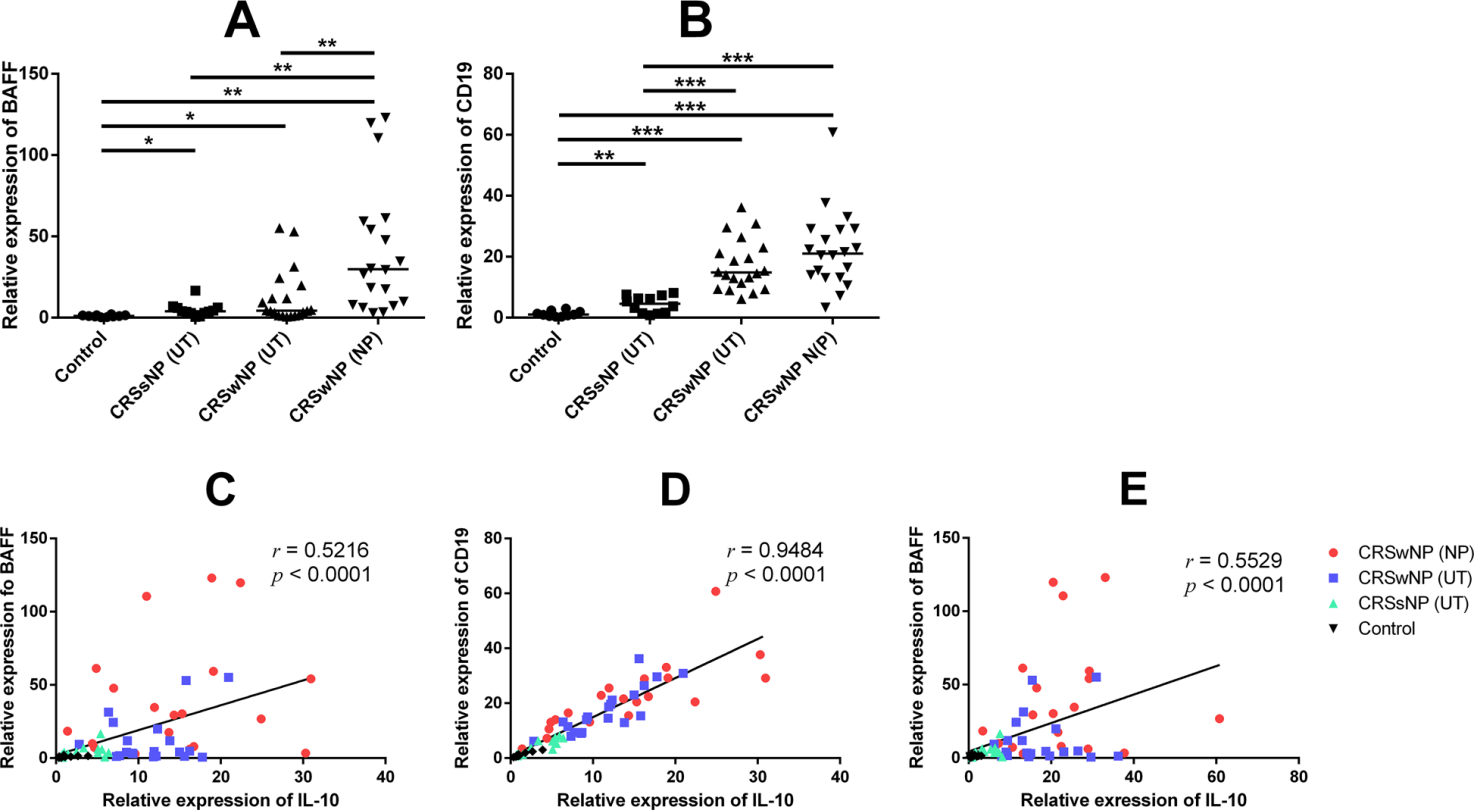
Influence of IL-10 expression on B cell population in the tissue. Expression of BAFF (A) and CD19 (B) mRNA among the groups. There was significant correlation in mRNA expression between IL-10 and BAFF (C) as well as between IL-10 and CD19 (D). CD19 and BAFF also showed significant correlation in their mRNA expression (E). * = p<0.05, ** = p<0.01, *** = p<0.001, r = Spearman’s rank correlation coefficient.

To investigate the influence of increased expression of IL-10 on B cell proliferation, expression of BAFF and CD19 mRNA were measured. Expression of BAFF and CD19 mRNA were significantly higher in the UT and NP of the CRSwNP group than in the UT of the control or CRSsNP groups (Fig 7A and 7B). In addition, there were significant correlations in the mRNA expression between IL-10 and BAFF (Fig 7C) as well as between IL-10 and CD19 (Fig 7D). CD19 and BAFF also showed significant correlation in their mRNA expression (Fig 7E).

### Expression of IL-10 and other inflammatory cytokines in murine polyp model

**Fig 8:**
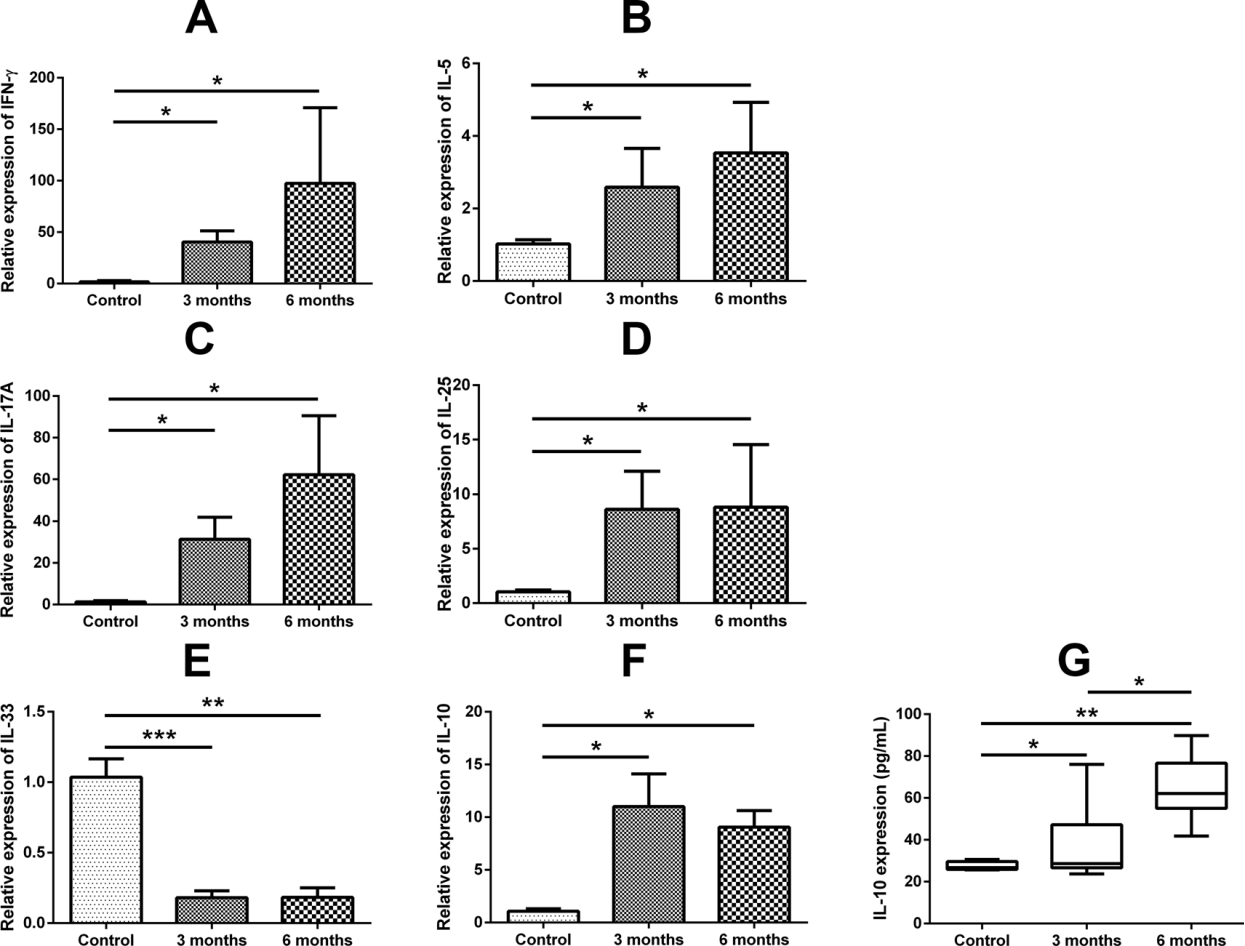
Expression of IL-10 and other cytokines in murine polyp model. Expressions of IFN-γ (A), IL-5 (B), IL-17A (C), IL-25 (D) and IL-10 (F) were significantly higher in the 3 months and 6 months murine polyp model than in control mice. Expression of IL-33 (E) was significantly lower in the 3 months and 6 months mouse model compared with the control mice. The protein level of IL-10 in the nasal lavage fluid was significantly higher in long-term CRS model groups compared with the control group (G). * = p<0.05, ** = p<0.01, *** = p<0.001.

Expressions of IFN-γ, IL-5, IL-17A, IL-25 and IL-10 were significantly higher in the 3 months and 6 months murine polyp model than in the control mice (Fig 8A–8D and 8F). Interestingly, similar to human tissue, the expression of IL-33 was significantly lower in the 3 months and 6 months mouse polyp model compared with the control mice (Fig 8E). As with the human samples, there was a significant positive correlation between the mRNA expression levels of IL-10 and those of IFN-γ, IL-5, IL-17A and IL-25 in the murine NP models. However, the mRNA expression levels of IL-10 and IL-33 showed a negative correlation (S4 Fig). To verify the protein expression levels of IL-10 in the murine model, the protein level of IL-10 in the nasal lavage fluid was evaluated. In the long-term CRS model, we observed a greatly increased expression of IL-10 compared with the control group (Fig 8G).

## Discussion

CRSwNP is a chronic mucosal inflammatory disease that involves complex pathogenesis and multiple etiologies. Previous studies have revealed that cells with immunosuppressive properties, such as regulatory T cells, were decreased in the polyp tissues of CRSwNP, compared to the mucosa of normal control or CRSsNP. The defects in these inhibitory responses were thought of as one of the key mechanisms in the pathogenesis of NPs [3,5]. However, suppressors of cytokine signaling (SOCS), which are known to regulate inflammatory cytokine activity, were increased in CRS mucosa, and this was interpreted as a response to the elevated levels of various cytokines in the already inflamed sinus mucosa [36]. In addition, we recently showed that IL-17C, which plays a key role in regulating the innate immune function of epithelial cells and providing a link between inflammation control and maintenance of the mucosal barrier function during infection, was upregulated in the polyp tissue of CRSwNP [37]. DNPCs were found to produce substantial amounts of IL-10, a potent anti-inflammatory cytokine that is essential for regulating the immune response, in response to staphylococcal exotoxin stimulation [12]. In our study, expression of IL-10 was also significantly higher in the tissues of CRSwNP (both UT tissue and NP) than in the tissue of the control or CRSsNP groups. As a result, IL-10 was thought to play a crucial role in polypogenesis, thus contributing to the pathogenesis of CRSwNP.

Several pathogens have evolved mechanisms that selectively up-regulate IL-10 during the course of an infection, presumably to create a more favorable microenvironment for the microbes [38]. For instance, TLR3 signaling regulates eosinophilia-associated cytokine production in CRSwNP via IL-10 production [39]. Staphylococcus aureus can produce various virulence factors or immunologic components such as microcapsules and toxins that contribute to the pathogenesis of CRSwNP [40–43]. Staphylococcal protein A (SpA) from S aureus coupled to immunoglobulins in immune complexes (ICs) induces the production of IL-10 and almost completely suppresses staphylococcal enterotoxin B-induced IL-5, IL-13, IFN-γ, and IL-17A production by DNPCs [44]. The up-regulated expression of IL-10 in the nasal mucosa or polyps in patients with CRSwNP can also suppress the production of pathogen related inflammatory cytokines, which in turn can facilitate immune avoidance and contribute to reduced pathogen elimination and may ultimately lead to chronic infection and inflammation in the tissue. In the present study, the signals for SpA were appreciably higher in the NPs of the CRWwNP group than in the UT of the CRSsNP or control groups. This result may be due to the up-regulation of IL-10, which leads to decreased pathogen clearance in the tissue of CRSwNP.

The relationship between expression of IL-10 and disease severity in patients with CRSwNP has also been recently reported. Patients with a more severe endotype of NP such as eosinophilic NP, or CRSwNP patients who had concomitant respiratory diseases such as bronchial asthma showed higher IL-10 expression levels in their blood or tissue samples, as opposed to patients who suffered a milder endotype of NP such as non-eosinophilc NP, or CRSwNP patients without any concomitant respiratory diseases [13–17,21,22,35,45–49]. Hossein Esmaeilzadeh *et al*. also demonstrated a correlation between the results of several subjective evaluations such as SNOT-22 and the IL-10 expression level in blood samples [35]. Because inflammatory molecules can often be potent activators of cell death, increasing levels of IL-10, which is induced in response to pathogens, can moderate the extent of apoptosis [8]. Therefore, in cases of parasitic infections or tuberculosis, IL-10 is a critical biomarker for poor disease outcome [50–52].

In the present study, the expression of IL-10 showed a positive correlation with the PNS CT score (Lund-Mackay CT score) in patients with CRSwNP. Because the Lund-Mackay CT score represents the severity of sinus inflammation, we believe that an increased expression of IL-10 in the tissues can also be an indicator of poor prognosis for medical and surgical treatment in patients with CRSwNP, though other inflammatory cytokines such as IFN-r, IL-5, IL-17A and IL-25 also correlate with the PNS CT score. Because numerous inflammatory mediators are activated in chronic mucosal inflammation [53], more complex analytic tools are needed to pinpoint the mechanisms underlying mucosal disease. In addition, further studies are needed to determine whether IL-10 can be used to indicate poor surgical outcome or increased polyp recurrence.

Imbalances in local Th1, Th2, Th17, and Treg responses appear to be involved in the pathogenesis of CRSwNP [3,5]. In the present study, although the mRNA expressions of IFN-γ, IL-5, and IL-17A were higher in the CRSwNP group than in the control or CRSsNP groups, the increase in expression levels were not as considerable as IL-10 or IL-25. This may be due to the immune suppressive function of IL-10 on other cytokines. The expression ratios of the selected inflammatory cytokines to IL-10 were calculated and the relative expressions of these inflammatory cytokines to IL-10 were significantly lower in the CRSwNP group. These results indicate that IL-10 may have a suppressive effect on Th1, Th2, and Th17 cytokines in the tissue of CRSwNP. The ratio of IL-25 to IL-10, however, was significantly increased in the CRSwNP group and both cytokines were co-localized in the same cells as was shown by immunofluorescent staining. Additional studies to verify the cells in which these cytokines were produced may be needed in the future.

A recent study proved that IL-25 secreted from the sinonasal epithelium plays a crucial role in the pathogenesis of CRSwNPs in Asian patients and suggested that mast cells may be the main source of IL-25 production [2]. Similarly, a higher expression ratio of IL-25 to IL-10 in the CRSwNP group in our study also indicated that IL-25 may be a crucial cytokine in the pathogenesis of CRSwNP. A strong positive correlation of some cytokines (IL-5, IFN-r and IL-17A) with IL-10 also indicated that expression of these cytokines are closely related with IL-10 expression and may be under the control of IL-10.

Interestingly, a well-documented distinction exists between the inflammation pattern of CRSwNP in Asians and Caucasians, where Asian patients with CRSwNP show Th1/Th17 dominance and neutrophilic activation, unlike the NP of Caucasians which are often associated with eosinophilic airway inflammation. This type of inflammation is frequently linked to comorbid asthma and activated T cells producing a Th2-biased cytokine profile. The inconsistency of the cytokine profiles of our study with those of other reports in the literature can be explained by these immunologic differences in CRSwNP between the two ethnic groups.

Because of the strong positive correlation between IL-10 and IL-25 and higher relative expression of IL-25 to IL-10 in the CRSwNP group, an in vitro stimulation of DNPCs with IL-25 and IL-10 was conducted. We found that IL-25 increased cytokine production, and IL-10 inhibited IL-25 induced cytokine production in the DNPCs. This result indicates that IL-25 can increase IL-10 expression as well as that of other inflammatory cytokines and that the immunosuppressive function of IL-10 is intact in the DNPCs of patients with CRSwNP.

IL-10 is known to down-regulate the expression of major histocompatibility complex (MHC) class II and co-stimulatory molecules of antigen presenting cells (APCs) leading to decreased T cell activation [54,55]. However, expression of the HLA-DRβ molecule was up-regulated in the tissues of patients with CRSwNP in our study, a result possibly due to increased infection signaling in the NPs. Monocytes and macrophages are the main sources of IL-10 in response to S. aureus in tissues [44]. In fact, the production of IL-10 by monocytes and macrophages is between 4 and 20 times higher than by DCs [56], and SpA, one important component of S aureus, showed a higher detection signal in NPs of CRSwNP than in the tissues of the control or CRSsNP groups. An earlier study revealed that macrophages from patients with CRSwNPs show reduced phagocytic capacity against S. aureus and were polarized toward a M2 phenotype compared to macrophages from patients with CRSsNPs [57].

BAFF-induced IgA production may be associated with eosinophils’ aggregation and degranulation, which aggravate tissue inflammation and ultimately causes polyp formation [58,59]. In contrast to its effect on cellular immunity, IL-10 functions as an activating factor that stimulates cell proliferation and immunoglobulin secretion of B lymphocytes. In our study, expression of both B cell activating factor and CD19 were significantly up-regulated in patients with CRSwNP and showed a strong positive correlation. In addition, IL-10 showed a significant positive correlation with BAFF or CD19. Therefore, IL-10 may be associated with B cells or may contribute to mucosal immunopathology in CRSwNP.

A previous study demonstrated that endogenous IL-10 is critical for the development of asthma-like responses in a murine asthma model wherein asthma was induced following i.p. sensitization and subsequent intranasal challenge with ovalbumin (OVA) [60]. In their study, IL-10 gene knockout (KO) mice showed significantly reduced IL-5 production, eosinophilic inflammation and mucus production without notable changes in IL-4 and IgE responses in comparison with wild-type controls [60]. In the present study, to strengthen the association between human and murine NP, we investigated the expression of IL-10 and other inflammatory cytokines in our previously described murine NP model [2,34]. After induction of an ovalbumin (OVA)-induced allergic rhinosinusitis (for 4 weeks), OVA with SEB was instilled into the nasal cavity of the mice for 8 weeks (3 months model) and 20 weeks (6 months model). Expressions of IL-10 and IL-17A were significantly up-regulated in the 3 months and 6 months models, compared with the control mice, indicating that IL-10 may play an important role in the pathogenesis of NP in the mouse NP model. Interestingly, IL-33 showed a similar expression pattern in human NPs. Future studies are needed to investigate the role of IL-10 in the pathogenesis of NP using IL-10 KO mice.

## Conclusion

Increased expression of IL-10, IL-10 related inflammatory cytokine, and IL-10 related B cell activation indicate that IL-10, a potent anti-inflammatory cytokine, has a pivotal role in the pathogenesis of CRSwNPs.

Further studies to evaluate the immunologic functions n of this cytokine in CRSwNP are needed in the future.

## Supporting Information

S1 Fig Constitutive cell and immune cell populations in DNPCs. The frequency of DNPCs expressing cytokeratin, vimentin, CD15/Singlec-8, CD4, CD8, CD79a, CD68, CD117, CD11c/CD303/HLA-DR, and CD1c/HLA-DR (CD11c^-^) were shown as mean with SEM.
(TIF)

S2 Fig Relative expression ratio of inflammatory cytokines to IL-10 among patient groups. Relative expression ratios of IFN-γ (A), IL-5 (B), IL-17A (C) and IL-25 (D) to IL-10 were shown as mean with SEM. ** = *p*<0.01, *** = *p*<0.001.
(TIF)

S3 Fig Correlations between Lund-Mackay CT scores and inflammatory cytokines. There were significant positive correlations between CT scores and expressions of other cytokines including IFN-γ (B), IL-17A (C) and IL-5 (D) and IL-25 (E), as well as IL-10 (A) in patients with CRSwNP. There were no significant correlations between the mRNA expression level of IL-10 and those of IL-4 (F) and IL-33 (G). *r* = *Spearman*’s rank correlation coefficient.
(TIF)

S4 Fig Correlations between IL-10 and inflammatory cytokine expressions in murine tissues. Significant positive correlations were determined between mRNA expression level of IL-10 and those of IFN-γ (A), IL-5 (B), IL-17A (C) and IL-25 (D) in murine NP models. There was a significant negative correlation between the mRNA expression level of IL-10 and IL-33 (E). *r* = *Spearman*’s rank correlation coefficient.
(TIF)

S1 Table Primer sequences used for real-time fluorescence qPCR. (DOCX)

S2 Table Anti-human antibodies used for flow cytometric analysis. (DOCX)
