# Correction: Role of Interleukin-10 on Nasal Polypogenesis in Patients with Chronic Rhinosinusitis with Nasal Polyps

**DOI:** 10.1371/journal.pone.0177755

**Published:** 2017-05-11

**Authors:** The PLOS ONE Staff

The image for Fig 8 is incorrectly duplicated in Fig 1. Please see the correct image for Fig 1 here. The publisher apologizes for the error.

**Fig 1:**
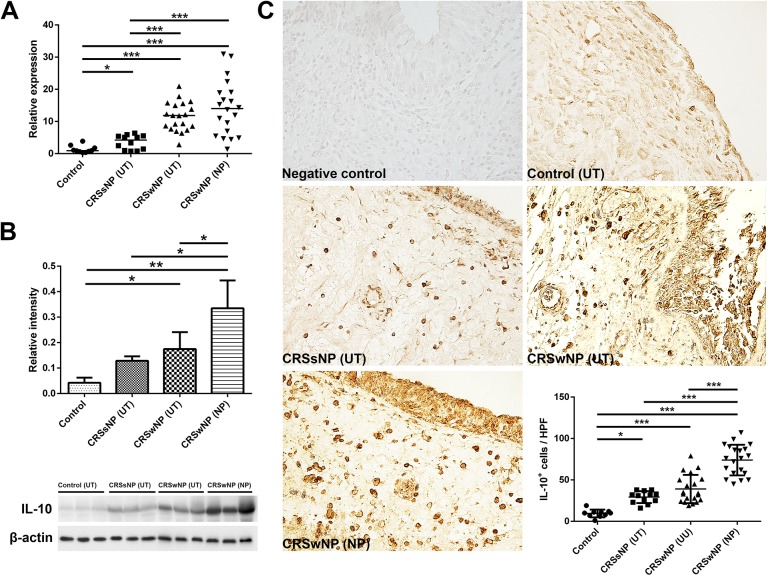
Expression of IL-10 among the different groups. A, mRNA expression of IL-10 in the UT tissue or NP tissue among groups. B, representative images of western blot assay and relative expression of IL-10 in the UT or NP among the groups. C, representative images of IL-10 immunohistochemistry and the number of IL-10 positive cells in the tissues from the different groups. CRSsNP, chronic rhinosinusitis without NP; CRSwNP, chronic rhinosinusitis with NP. * = *p*<0.05, ** = *p*<0.01, *** = *p*<0.001.

## References

[1] Xu J, Han R, Kim DW, Mo J-H, Jin Y, Rha K-S, et al (2016) Role of Interleukin-10 on Nasal Polypogenesis in Patients with Chronic Rhinosinusitis with Nasal Polyps. PLoS ONE 11(9): e0161013 doi:10.1371/journal.pone.0161013 27584662 10.1371/journal.pone.0161013 PMC5008817

