# Supplementary figures and images for: Role of Interleukin-10 on Nasal Polypogenesis in Patients with Chronic Rhinosinusitis with Nasal Polyps
Source: PLoS One. 2016 Sep 1;11(9):e0161013. doi: 10.1371/journal.pone.0161013 (PMC5008817; doi:10.1371/journal.pone.0161013)

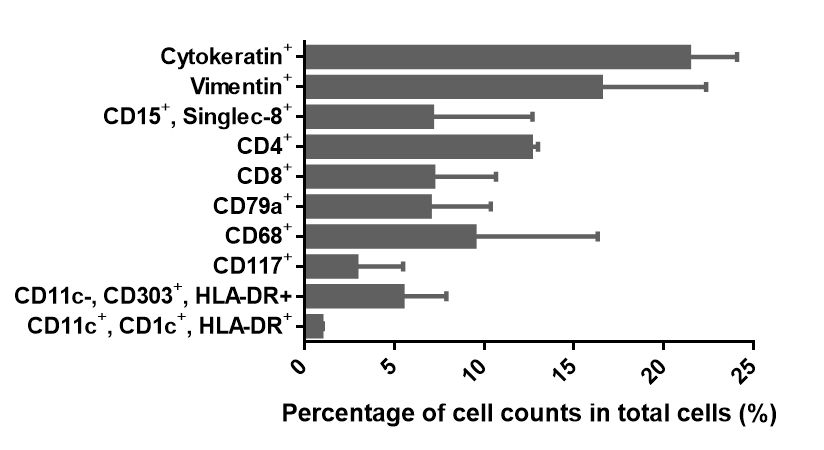

Supplement: S1 Fig — The frequency of DNPCs expressing cytokeratin, vimentin, CD15/Singlec-8, CD4, CD8, CD79a, CD68, CD117, CD11c/CD303/HLA-DR, and CD1c/HLA-DR (CD11c-) were shown as mean with SEM. (TIF) [file pone.0161013.s001.tif]

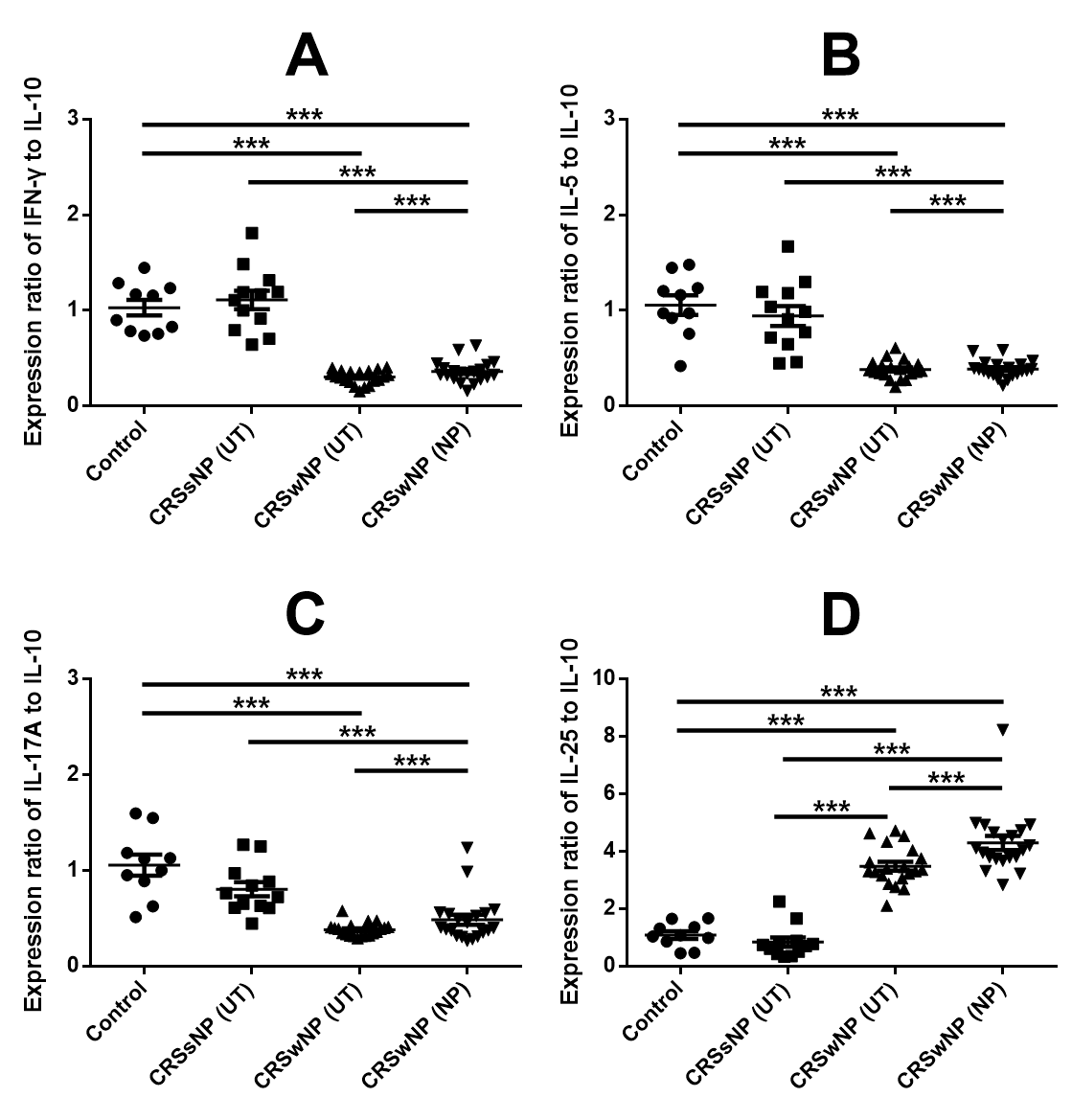

Supplement: S2 Fig — Relative expression ratios of IFN-γ (A), IL-5 (B), IL-17A (C) and IL-25 (D) to IL-10 were shown as mean with SEM. ** = p<0.01, *** = p<0.001. (TIF) [file pone.0161013.s002.tif]

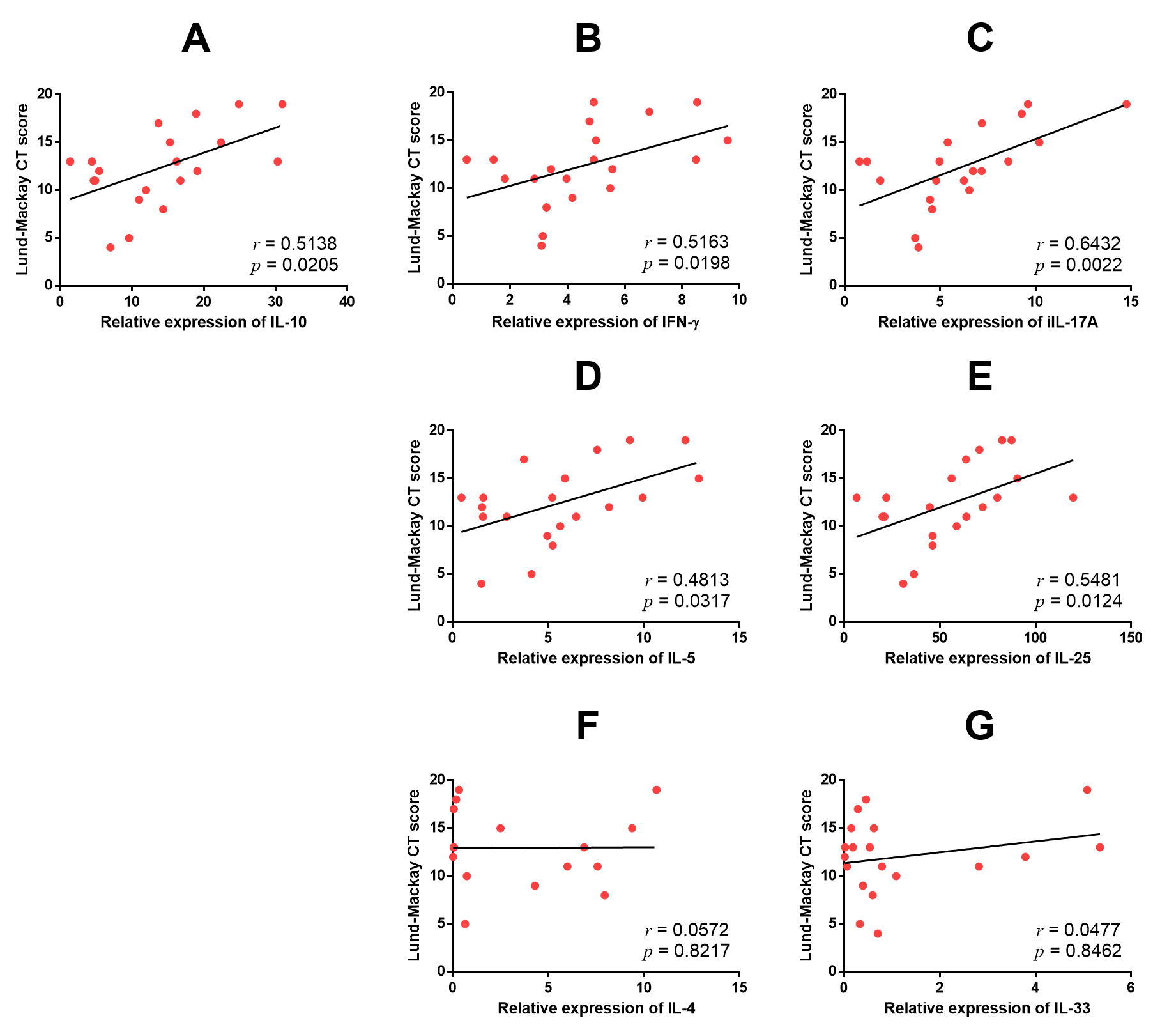

Supplement: S3 Fig — There were significant positive correlations between CT scores and expressions of other cytokines including IFN-γ (B), IL-17A (C) and IL-5 (D) and IL-25 (E), as well as IL-10 (A) in patients with CRSwNP. There were no significant correlations between the mRNA expression level of IL-10 and those of IL-4 (F) and IL-33 (G). r = Spearman’s rank correlation coefficient. (TIF) [file pone.0161013.s003.tif]

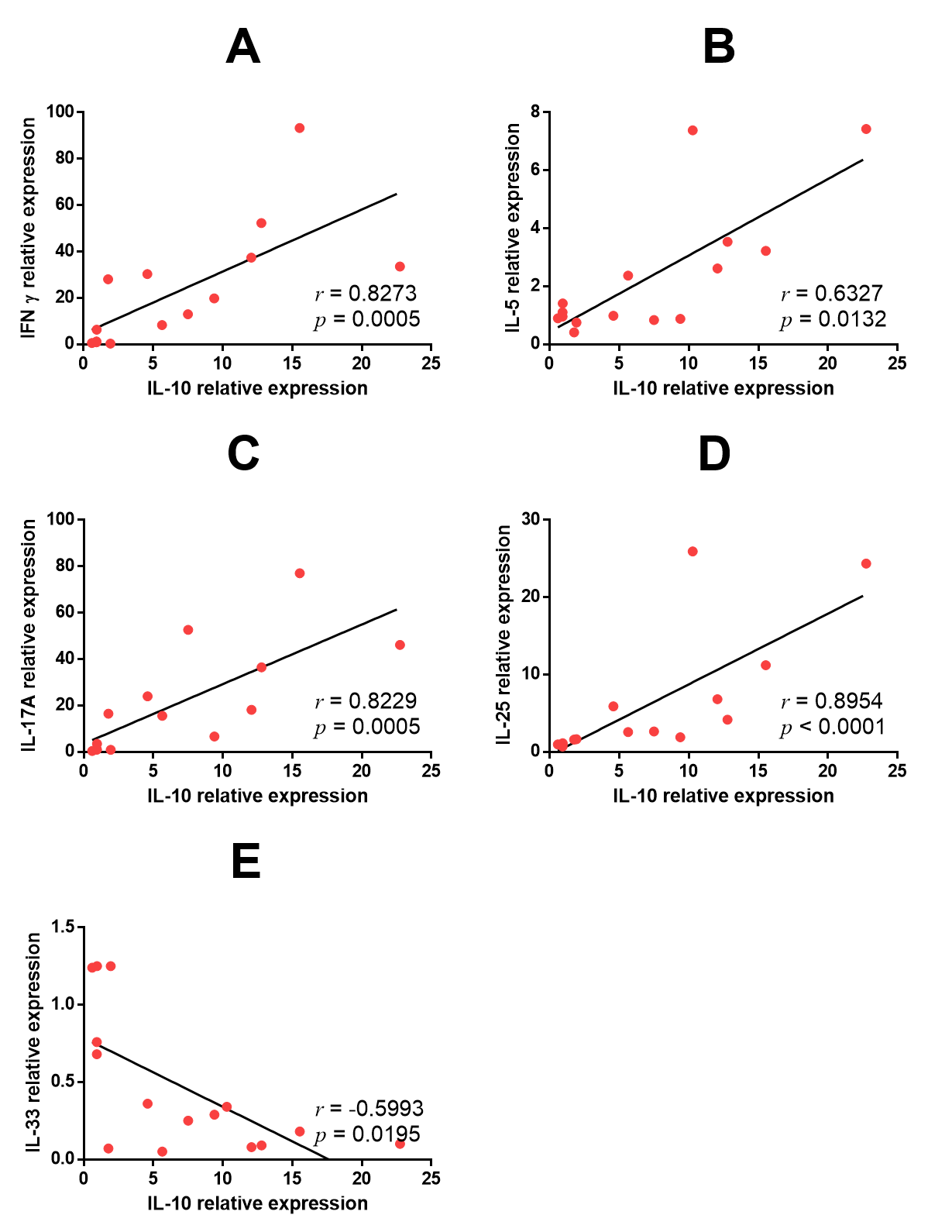

Supplement: S4 Fig — Significant positive correlations were determined between mRNA expression level of IL-10 and those of IFN-γ (A), IL-5 (B), IL-17A (C) and IL-25 (D) in murine NP models. There was a significant negative correlation between the mRNA expression level of IL-10 and IL-33 (E). r = Spearman’s rank correlation coefficient. (TIF) [file pone.0161013.s004.tif]
