# Supplementary material for: Role of Interleukin-10 on Nasal Polypogenesis in Patients with Chronic Rhinosinusitis with Nasal Polyps
Source: PLoS One. 2016 Sep 1;11(9):e0161013. doi: 10.1371/journal.pone.0161013 (PMC5008817; doi:10.1371/journal.pone.0161013)
Supplement: S1 Table — (DOCX) [file pone.0161013.s005.docx]

| **S1 Table. Primer sequences used for real-time fluorescence qPCR.** | | | | | |
| --- | --- | --- | --- | --- | --- |
| Organism | Molecules | Forward primer sequence (5’->3’) | Reverse primer sequence (5’->3’) | Product size (bp) | Genbank accession number |
| Homo Sapiens | GAPDH | AAATCAAGTGGGGCGATGCT | CAAATGAGCCCCAGCCTTCT | 86 | NM_002046.5 |
| Homo Sapiens | IFN-γ | AGTGATGGCTGAACTGTCGC | CTGGGATGCTCTTCGACCTC | 85 | NM_000619.2 |
| Homo Sapiens | IL-4 | GTGCACCGAGTTGACCGTAA | GCGAGTGTCCTTCTCATGGT | 124 | NM_000529.3 |
| Homo Sapiens | IL-5 | GGATGCTTCTGCATTTGAGTTT | CAGTGCCAAGGTCTCTTTCA | 104 | NM_000879.2 |
| Homo Sapiens | IL-10 | GGCACCCAGTCTGAGAACAG | ACTCTGCTGAAGGCATCTCG | 86 | NM_000572.2 |
| Homo Sapiens | IL-17A | CCCCATCCAGCAAGAGATCC | CCCACGGACACCAGTATCTT | 90 | NM_002190.2 |
| Homo Sapiens | IL-25 | GTCATGGGAACCCACACCTA | TGCTGTTGAGGGGTCCATCT | 175 | NM_022789.3 |
| Homo Sapiens | IL-33 | CATGCCAACAACAAGGAACA | AGGACAAAGAAGGCCTGGTC | 77 | NM_033439.3 |
| Homo Sapiens | CD19 | GATAACGCTGTGCTGCAGTG | AAGGGTTTAAGCGGGGACTC | 89 | NM_001770.5 |
| Homo Sapiens | BAFF | TGCAGACAGTGAAACACCAAC | GACATGGACCTTCTTCCTCTGA | 208 | NM_006573.4 |
| Homo Sapiens | HLA-DRα | CCTGACCAATCAGGCGAGTT | GTTGGCCAATGCACCTTGAG | 141 | NM_019111.4 |
| Homo Sapiens | HLA-DRβ1 | GTTTCCTGTGGCAGCCTAAGA | TTCCAGTACTCAGCGTCAGG | 169 | NM_002124.3 |
| Homo Sapiens | TGF β1 | AAATTGAGGGCTTTCGCCTTA | GAACCCGTTGATGTCCACTTG | 80 | NM_000660.5 |
| Homo Sapiens | MMP9 | TCTATGGTCCTCGCCCTGAA | CATCGTCCACCGGACTCAAA | 219 | NM_004994.2 |
| Homo Sapiens | TIMP1 | CGCAGCGAGGAGTTTCTCAT | GCAGTTTGCAGGGGATGGAT | 184 | NM_003254.2 |
| Mus musculus | GAPDH | CTTCAACAGCAACTCCCACTC | GCCGTATTCATTGTCATACCA | 103 | NM_008084.3 |
| Mus musculus | IFN-γ | GCTACACACTGCATCTTGGC | GGCTTTCAATGACTGTGCCG | 82 | NM_008337.4 |
| Mus musculus | IL-5 | GGGGGTACTGTGGAAATGCT | AATCCAGGAACTGCCTCGTC | 112 | NM_010558.1 |
| Mus musculus | IL-10 | TAACTGCACCCACTTCCCAG | AAGGCTTGGCAACCCAAGTA | 165 | NM_010548.2 |
| Mus musculus | IL-17A | TCAAAGCTCAGCGTGTCCAA | CGTGGAACGGTTGAGGTAGT | 133 | NM_010552.3 |
| Mus musculus | IL-25 | ACCACAACCAGACGGTCTTC | AGCCAAGGAGACTCGGTAGA | 101 | NM_080729.3 |
| Mus musculus | IL-33 | CCGTTCTGGCCTCACCATAA | GACGCAGCAAATGCTTGGAT | 165 | NM_133775.2 |
