# Supplementary material for: Role of Interleukin-10 on Nasal Polypogenesis in Patients with Chronic Rhinosinusitis with Nasal Polyps
Source: PLoS One. 2016 Sep 1;11(9):e0161013. doi: 10.1371/journal.pone.0161013 (PMC5008817; doi:10.1371/journal.pone.0161013)
Supplement: S2 Table — (DOCX) [file pone.0161013.s006.docx]

| **S2 Table. Anti-human antibodies used for flow cytometric analysis.** | | | | | |
| --- | --- | --- | --- | --- | --- |
| Antibody | Cell type | Clone | Isotype | Dilution | Conjunct fluorescence |
| Anti-Cytokeratin antibody | Epithelial cell | CK3-6H5 | mouse IgG1 | 1:100 | FITC |
| Anti-Vimentin antibody | Fibroblast | REA409 | mouse IgG1 | 1:100 | FITC |
| Anti-CD15 antibody | Eosinophil | VIMC6 | mouse IgM | 1:100 | PE |
| Anti-Singlec-8 antibody | Eosinophil | 7C9 | mouse IgG1k | 1:100 | APC |
| Anti-CD4 antibody | T helper cell | M-T466 | mouse IgG1 | 1:100 | FITC |
| Anti-CD8 antibody | Cytotoxic T cell | BW135/80 | mouse IgG2a | 1:100 | FITC |
| Anti-CD79a antibody | B cell | HM47 | mouse IgG1k | 1:100 | PE |
| Anti-CD68 antibody | Macrophage | Y1/82A | mouse IgG2b | 1:100 | FITC |
| Anti-CD117 antibody | Mast cell | A3C6E2 | mouse IgG1 | 1:100 | PE |
| Anti-HLA-DR antibody | Antigen presenting cell | AC122 | mouse IgG2a | 1:100 | APC |
| Anti-CD11c antibody | Pan dendritic cell | MJ4-27G12 | mouse IgG2b | 1:100 | FITC |
| Anti-CD303 (BDCA-2) antibody | Plasmacytoid dendritic cell | AC144 | mouse IgG1 | 1:100 | FITC |
| Anti-CD1c (BDCA-1) antibody | Myeloid dendritic cell | AD5-8E7 | mouse IgG2a | 1:100 | PE |
